# Case report: Persistent Müllerian duct syndrome and enlarged prostatic utricle in a male dog

**DOI:** 10.3389/fvets.2023.1185621

**Published:** 2023-07-04

**Authors:** Peter J. Welsh, Kaylyn McDaniel, Elizabeth W. Goldsmith, Joshua D. Ramsay, Alan Conley, Tina Jo Owen, Yoko M. Ambrosini, Michela Ciccarelli

**Affiliations:** ^1^Department of Veterinary Clinical Sciences, Washington State University, Pullman, WA, United States; ^2^Department of Veterinary Microbiology and Pathology, Washington State University, Pullman, WA, United States; ^3^Washington Animal Disease Diagnostic Laboratory, Washington State University, Pullman, WA, United States; ^4^Pathology Services, North American Science Associates, Minneapolis, MN, United States; ^5^School of Veterinary Medicine, University of California, Davis, Davis, CA, United States

**Keywords:** uterus masculinus, disorder of sex development, hematuria, urometra, prostatic utricle

## Abstract

A 1-year-old male intact Miniature Schnauzer mix was presented for chronic intermittent hematuria. Abdominal ultrasonography revealed a large, fluid-filled cystic structure extending cranially and dorsally to the prostate. Computed tomography scan images revealed that the fluid-filled cavity resembled a uterus, with both horns entering the scrotum through the inguinal canal adjacent to the testes. On cytogenetic analysis, the dog was found to have a homozygote mutation on *AMHRII* consistent with persistent Müllerian duct syndrome (PMDS). A gonadohysterectomy was performed, and surgical and histologic findings confirmed the presence of a uterus, oviducts, vagina, and testes in this dog. Additionally, an intraoperative fluoroscopy exam revealed a communication between the uterus and the bladder via an enlarged utricle, explaining the hematuria and urine in the reproductive tract (urometra). To our knowledge, this is the first clinical report of a phenotypically intact male dog with PMDS and urometra due to an enlarged prostatic utricle. This case illustrates a combination of a disorder of sex and urogenital sinus development.

## 1. Background

Disorders of sexual differentiation in mammals are classified into three categories: chromosomal, gonadal, and phenotypical. Persistent Müllerian duct syndrome (PMDS) is a phenotypical disorder of sex development characterized by not only XY chromosomes, testes, and male genitalia but also oviducts, uterus, cervix, and cranial vagina. PMDS is considered an inherited homozygous recessive male pseudohermaphroditism, diagnosed most commonly in humans (1) and in familiarly predisposed Miniature Schnauzers (2). There are other reports of this syndrome in cats (3), goats (4, 5), bison (6), and beavers (7). The persistence of Müllerian ducts as a remnant uterus, dorsal to the prostate, bifurcating and extending cranial to the epididymis, is a normal finding in male giant anteaters (8). All reported clinical cases of PMDS are due to mutations in genes encoding Anti-Müllerian Hormone (AMH) and/or AMH receptors (AMHR) (2, 9–14). In the Miniature Schnauzer, PMDS is associated with a C to T transition in exon 3 of the Müllerian hormone type II receptor (*AMHRII)* (14, 15).

During embryonic development, the presence of the Y chromosome and the *Sry* gene determine the development of the genital ridges to the testes of many mammals. The Leydig cells produce testosterone, which is responsible for the development of the mesonephric duct into the male reproductive duct system. Sertoli cells produce an anti-Müllerian hormone, which causes the regression of the Müllerian (paramesonephric) ducts, which would otherwise develop into oviducts, the uterus, and the cranial portion of the vagina. In the female embryo, the lack of the Y chromosome, *Sry*, and testosterone, together with the expression of FoxL2, among other transcription factors leads to the development of the ovaries and the tubular female reproductive tract. After the chromosomal and the gonadal differentiation, any abnormalities of AMH production or its receptor can lead to the development of the female reproductive tract in an otherwise normal male. Males affected by PMDS are often cryptorchids since the presence of a uterus interferes with normal testicular descent (12, 16). In some cases, the persistent Müllerian ducts can be pulled through the inguinal canal leading to inguinal hernias (17).

This condition may be associated with other abnormalities, such as congenital prostatic cysts and enlarged prostatic utricles, as presented in this case. The normal prostatic utricle is a small, blind opening at the level of the colliculus seminalis. It is the male homolog of the female uterus and the vagina. It has no specific function (18). However, fluid accumulation can lead to cystic formation and secondary clinical signs such as constipation, urinary retention, hematuria, urinary incontinence, recurrent urinary tract infections, and obstructive azoospermia (19). In stallions presented for ejaculatory problems, it is relatively common to find cysts at the level of the colliculus seminalis that can obstruct the ampullae of the deferent ducts. These cysts are reported to be remnants of the Müllerian ducts (20). Two kinds of Müllerian duct remnants resulting in midline cystic structures have been reported in humans: enlarged prostatic utricles that communicate with the urethra and do not communicate with Müllerian duct cysts. Enlarged prostatic utricles are considered congenital and are often observed in boys affected by intersex, hypospadias (11–14%), and cryptorchidism (21). The embryologic origin of the utricle is still under debate. The utricle appears to have a dual origin of an admixture of the urogenital sinus, Wolffian cells caudally, and Müllerian cells cranially (22). Utricular abnormalities have been commonly discovered in the incomplete regression of Müllerian duct cases or the incomplete closure of the urogenital sinus caused by an error in production or sensitivity to AMH (21). This is likely the case with the dog presented in the case report.

Several cases of prostatic cystic dilations communicating with the urethra and containing urine have been described in dogs (23, 24). However, none of the cases have been associated with PMDS and urometra, as in this case. Additionally, this is the first case describing an intact male naturally affected by PMDS with bilaterally descended testes demonstrating histologically normal spermatogenesis. This case report concerns an intact male Miniature Schnauzer mix presented for intermittent hematuria, in which PMDS, urometra, and enlarged prostatic utricle were discovered and surgically corrected.

## 2. Case presentation

A 1-year-old male intact Miniature Schnauzer mix was presented to the primary veterinarian in March 2022 with lethargy and a blood-tinged discharge from his prepuce following urination. A free-catch urinalysis was performed, revealing no bacteria, and a urine-specific gravity of 1.030 was determined. An abbreviated abdominal ultrasound revealed a suspected prostatic cyst or enlargement. Treatment with finasteride (2 mg PO q24h) and enrofloxacin (68 mg PO q24h for 30 days) was initiated. The owner discontinued enrofloxacin as it appeared ineffective and continued the finasteride as it seemed to decrease the blood-tinged discharge significantly. Small amounts of blood were noted in the patient's urine, predominately in the morning, and drops of blood were noted from the prepuce throughout the day. A referral was recommended due to the lack of clinical resolution.

The patient was presented to the Washington State University Veterinary Teaching Hospital's Small Animal Internal Medicine service 3 months following the onset of clinical signs. The owner reported no change in the previously reported clinical signs. No urinary accidents or incontinence were noted, and the patient was otherwise healthy. On physical examination, the patient's vital parameters were within normal limits. The testicles were descended and normal on palpation. Upon initial examination, the penis was extruded and revealed no masses or active discharge. However, on a recheck 2 days later, hemorrhagic discharge from the prepuce was observed. The remainder of the patient's physical examination was within normal limits. Initial diagnostic testing included a complete blood count (CBC), serum biochemical profile, urinalysis, urine culture, and focal ultrasonographic evaluation of the urinary system. The CBC revealed a mild macrocytosis (78 fL; reference interval, 62–73 fL) and a mild increase in MCH (27 pg; reference interval, 22–26 pg). Serum biochemistry identified mild hypocholesterolemia (129 mg/dL; reference interval, 134–359), hyponatremia (147 mEq/L; reference interval, 149–158), and hypochloremia (111 mEq/L, reference interval, 112–119). A cystocentesis sample revealed amber-colored urine with a specific gravity >1.035 with 1+ protein and 3+ blood. Sterile pyuria was present with 16–30 white blood cells per high-power field. The urine culture was negative.

**Figure 1:**
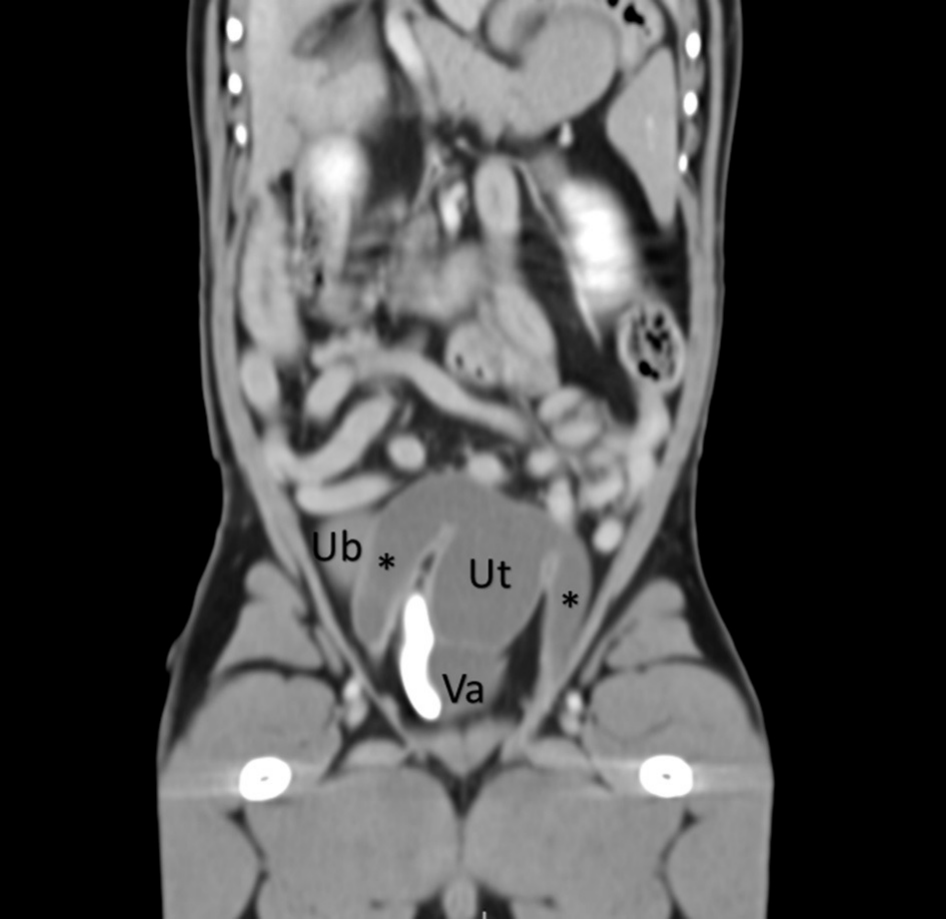
A coronal abdominal CT post-contrast reveals a uterus (Ut) and vagina (Va) dorsal to the urinary bladder (UB) and ventral to the colon. A contrast-filled urethra is also visualized to the right of the vagina. The uterine horns are visualized extending caudally (asterisks).

Focal urinary ultrasonography revealed a markedly large, tubular structure arising from the left dorsal aspect of the prostate extending cranial adjacent to the urinary bladder measuring approximately 7 cm × 4 cm × 2 cm. Hypoechoic fine echogenic material and several large, irregular accumulations of loosely structured echogenic material were noted within the structure. The remainder of the prostatic parenchyma was normal in echogenicity, size, and margination. A small volume of a gravity-dependent, echogenic material was appreciated within the urinary bladder. The testicles appeared normal; however, a small volume of hypoechoic effusion was noted within the scrotal sac and several hyperechoic, mild-to-moderate shadowing foci. The kidneys and ureters were within normal limits. An abdominal CT (Toshiba CGGT-018A) with single-phase angiography was acquired for further evaluation and surgical planning. A complex, lobulated cystic structure arising from the right dorsal margin of the prostate was present (Figure 1). Bilaterally symmetric tubular fluid-filled structures followed parallel with the spermatic cords extending from the level of the testicles through the inguinal ring, inserting caudal into the paraprostatic structure (Figure 1, Supplementary Video 1). Several small gravity-dependent foci were appreciated within the tubular structures. The urinary bladder revealed no apparent connection to the paraprostatic structure with contrast excretion from the ureters into the bladder only. The remainder of the abdomen was unremarkable. An ultrasound-guided fine needle aspirate of the paraprostatic structure revealed grossly hemorrhagic fluid with a cell count of 17, 710 nucleated cells/uL, a packed cell volume of 4%, and a total protein of 1.8 g/dL. Cytology was consistent with moderate neutrophilic inflammation with segmented and mildly degenerate neutrophils with no apparent bacterial organisms or neoplastic cells. The creatinine concentration of the fluid was 13.9 mg/dL.

Persistent Müllerian duct syndrome was suspected, given the imaging findings and the miniature schnauzer breed combination. Endocrine testing and molecular genetic analysis for PMDS were performed. Serum endocrine analysis submitted to the University of California-Davis revealed an inhibin B level of 57.6 pg/mL (no canine male reference range), a progesterone level of 0.18 ng/mL (no canine male reference range), a testosterone level of 212.8 pg/mL, and an anti- Müllerian hormone level of 196 ng/mL. The typical intact canine male reference range for testosterone is >1, 000 pg/mL; the cryptorchid male reference range is 100–500 pg/mL, and the castrated male reference range is <50 pg/mL. The Anti-Müllerian hormone level is considered 3–4 times higher than expected for normal fertile males. Molecular genetic analysis (Paw Print Genetics, Spokane, WA) identified two mutant copies of the DNA sequences for *AMHRII*, indicating that the patient was at risk of or affected by PMDS. Exploratory laparotomy was recommended due to the conglomeration of diagnostics findings in this patient.

### 2.1. Surgical procedure

The patient presented for exploratory laparotomy 2 months following the diagnostic workup. Dexmedetomidine (Dexdomitor^®^ Zoeits Inc., Parsippany, NJ; 5mcg/kg, IM), methadone (Akorn Inc., Lake Forest, IL; 0.2 mg/kg, IM), propofol (Propoflo Zoeits Inc., Parsippany, NJ; 3 mg/kg titrated, IV), and isoflurane were used as premedication, induction, and maintenance agents. Cefazolin (Apotex Corp., Weston, FL; 22 mg/kg, IV) was given 30 min prior to the incision and every 90 min thereafter. A continuous intravenous infusion of PlasmaLyte (pHyLyte^®^ Dechra Pharmaceuticals plc, Northwich, England) was administered.

**Figure 2:**
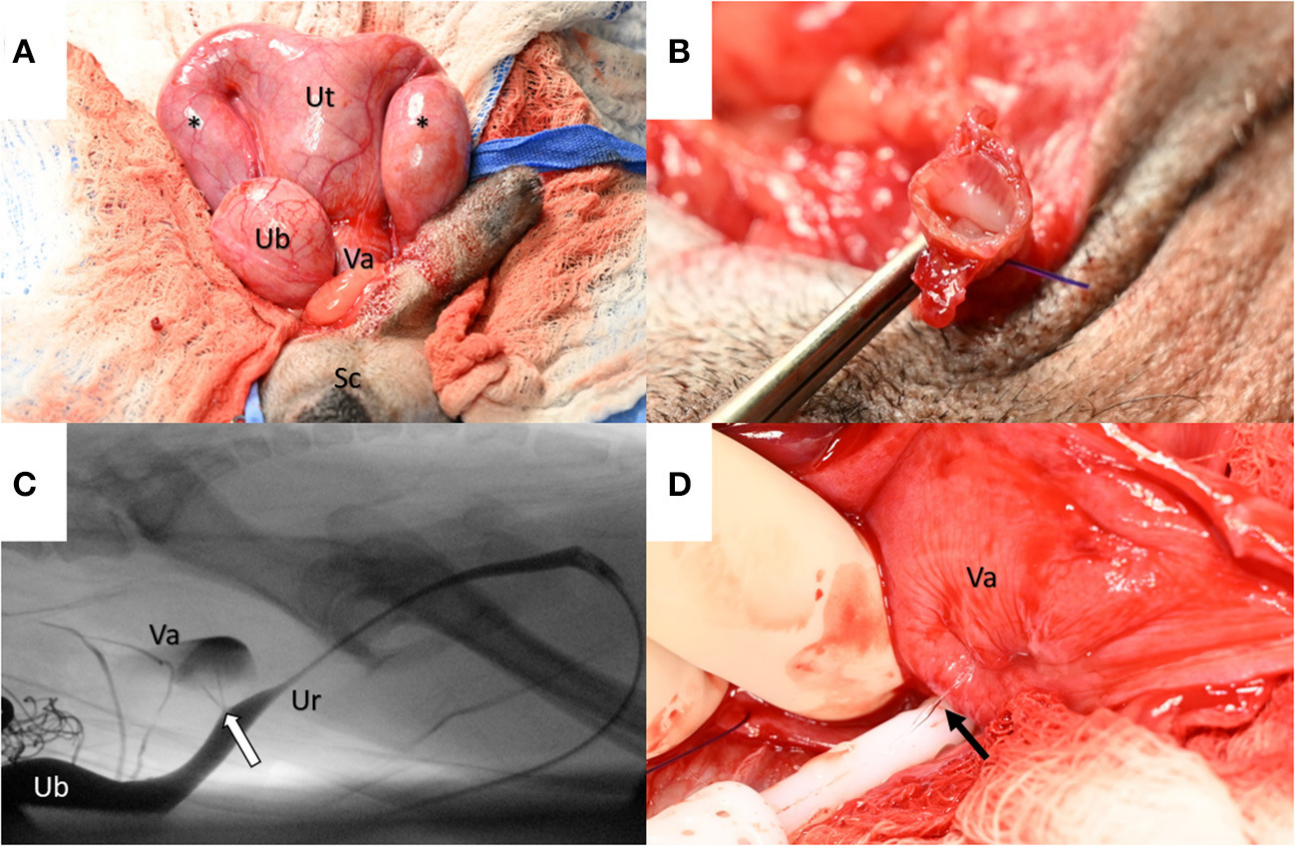
**(A)** Intraoperatively, the uterus (Ut) was identified as dorsal to the urinary bladder (UB) with two fluid-filled horns (asterisks) that extended through the inguinal rings into the scrotum (Sc). A rudimentary cervix delineated the uterus from the vagina (Va). **(B)** The uterine horns were found to envelop the spermatic cords following excision. **(C)** An intraoperative fluoroscopic contrast cystourethrogram identified the prostatic utricle (arrow) as a communication between the prostatic urethra (Ur) and the vagina (Va) (Note: to the left side of the image dorsal to the urinary bladder (UB), a radiopaque surgical sponge is observed). **(D)** The vagina (Va) was dissected to identify and occlude communication with the prostatic urethra. With a digital expression of the urinary bladder, communication was evident between the vagina and prostatic urethra via a urine stream (arrow).

A standard midline laparotomy was performed. The uterus was identified with two moderately distended fluid-filled horns extending toward the inguinal rings (Figure 2A). The uterine body had a thin membranous band (suspected rudimentary cervix), demarcating a division between the uterine body and the vagina before continuing down to the level of the prostate (Figure 2A). The prostate was identified along the ventral neck of the bladder and was grossly normal in appearance and palpation. The remainder of the abdominal exploration was unremarkable.

The testes were exteriorized from the scrotum via a prescrotal incision and excised via the 3-clamp technique using encircling and transfixing ligatures of 3–0 polydioxanone suture (PDS^®^II; Ethicon Inc., Rariton, NJ). The testes and spermatic cord were enveloped within the descending uterine horns tracing through the inguinal canal (Figure 2B).

Uterine fluid was collected for cytology and culture. The uterine arteries at the level of the body were ligated with 3–0 PDS^®^II and transected. The uterine body was ligated with 3–0 PDS^®^II with one encircling and one transfixing ligature cranial to the rudimentary cervix. The uterine body was excised using Metzenbaum scissors and submitted for histopathology. The uterine/vaginal stump was later found to distend with a digital expression of the urinary bladder, suggesting an enlarged prostatic utricle. A size 8 French Foley urinary catheter was placed intraoperatively. Under fluoroscopic guidance, a contrast cystourethrogram evidenced two small ostia at the level of the colliculus seminalis that communicated with the uterine/vaginal stump (Figure 2C, Supplementary Video 2).

The dorsal aspect of the stump was sharply incised distally, and the bladder was infused with sterile saline and expressed, allowing gross visualization of the Ostia (Figure 2D). A purse-string-like transfixing suture was placed, incorporating both ostia collectively with 3–0 PDS^®^II. The suture was leak-pressure tested with sterile saline infused via the Foley catheter. The dorsal incision within the stump was sutured with 3–0 PDS^®^II in a simple continuous pattern to the level of the previously placed uterine artery ligatures. The remaining cranial portion of the uterine/vaginal stump was excised and submitted for histopathology. The abdomen was lavaged, instruments were changed, and the sponge count was confirmed. Omentalization of the uterine stump was facilitated by packing and suturing the omentum circumferentially with two simple continuous patterns of 3–0 PDS^®^II. Liposomal bupivacaine (Nocita^®^ Elanco Animal Health, Greenfield, IN; 5.3 mg/kg) was infiltrated into the peri-incisional subcutaneous tissue with a 25-gauge needle. The abdominal incision was routinely closed.

Postoperatively, the dog recovered under close monitoring. He was maintained on a continuous intravenous infusion of PlasmaLyte (2 mLs/kg/h). Other postoperative medications included methadone (0.2 mg/kg IV every 6 h as needed), carprofen injection (Rimadyl^®^ Zoeits Inc., Parsippany, NJ, 2.2 mg/kg SQ once), carprofen tablets (Rimadyl^®^ 2.6 mg/kg PO q12), and gabapentin capsules (Westminster Pharmaceuticals LLC, Nashville, TN; 13.9 mg/kg PO q12). No postoperative or anesthetic complications were noted, and the patient was discharged the following day.

### 2.2. Epididymal semen extraction and evaluation

After castration, the testicles were collected, and the epididymides were isolated. The epididymal tails were excised and placed in a Petri dish. A slicing float-up technique was used to recover the sperm into the semen extender. The sample was filtered to remove epithelial cells and evaluated by light microscopy and Computer Assisted Sperm Analysis (CASA, SpermVision^®^). The concentration was low and could not be assessed by CASA; few spermatozoa were motile, and most of the spermatozoa showed proximal droplets, a common finding in the sperm extracted from the epididymis. The fertility of the dog could not be determined.

### 2.3. Histopathology and bacteriology

**Figure 3:**
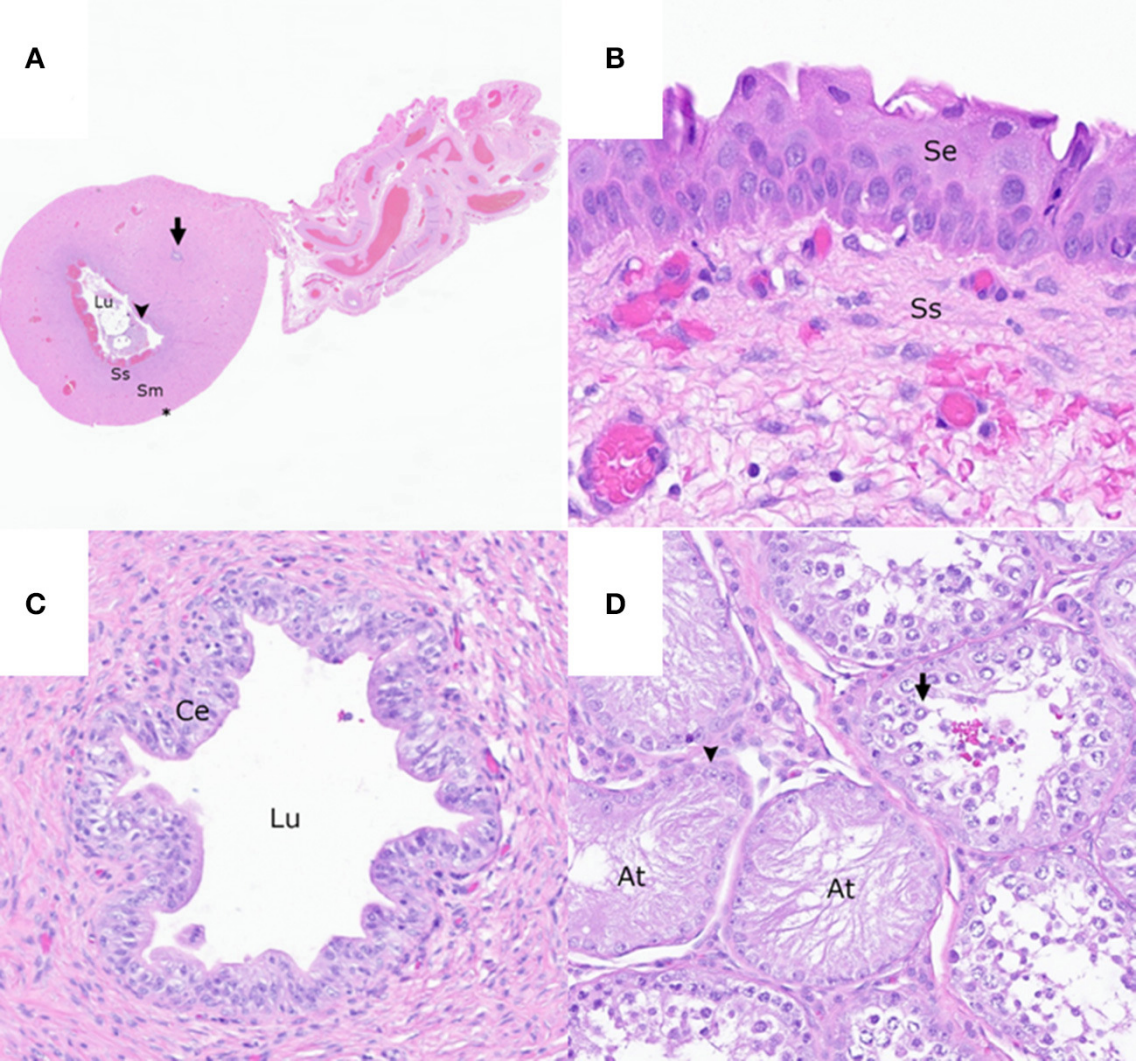
The reproductive tract of the patient. **(A)** Cross-section of the rudimentary uterine horn consisting of an epithelial-lined (arrowhead) lumen (Lu) containing necrotic cellular debris, a subepithelial stroma (Ss), smooth muscle layers (Sm), a serosa (asterisk), and an intramuscular rudimentary ductus deferens (arrow). Hematoxylin and eosin (HE). **(B)** Stratified squamous epithelium (Se) and loose collagenous subepithelial stroma (Ss) of the rudimentary vaginal tissue HE. **(C)** Rudimentary ductus deferens with a lumen (Lu) lined by a pseudostratified columnar epithelium (Ce) HE. **(D)** Testis with multifocal atrophied seminiferous tubules (At) lined by a single layer of Sertoli cells (arrowhead) and the seminiferous tubules with germinal epithelial degeneration characterized by a disorganized arrangement of germ cells (arrow) HE.

Surgically excised tissues were submitted to the Washington Animal Disease Diagnostic Laboratory (WADDL) for assessment. According to WADDL standard operating procedures, the submitted tissues were formalin-fixed, processed, and stained with hematoxylin and eosin. On histologic examination, the rudimentary uterine body and uterine horns (uterus masculinus) consisted of tubular structures (Figure 3A) lined by round to polygonal to columnar epithelial cells, multinucleated giant cells, and multifocal ulceration. The lumen contained necrotic debris, hemorrhage, and a few to numerous macrophages. The rudimentary vagina was lined by a one to five-cell thick, stratified, polygonal to squamous epithelium (Figure 3B). Deep to the epithelium, the rudimentary uterine body, uterine horns, and vagina were composed of a loose collagenous subepithelial stroma, an inner circumferential layer of smooth muscle, an outer longitudinal layer of smooth muscle, and serosa (Figure 3A).

Cross-sections of rudimentary ductus deferens were randomly interspersed throughout the smooth muscle layers of the rudimentary uterus (Figures 3B, C) and the vagina and within the connective tissue adjacent to the right testis and epididymis. These structures were lined by ciliated and non-ciliated columnar epithelial cells. Within the left and right testes, <10% of the seminiferous tubules had evidence of complete spermiogenesis, and <10% of the seminiferous tubules were atrophied and lined by a single layer of Sertoli cell (Figure 3D). The remaining seminiferous tubules had evidence of germinal epithelial degeneration with a disorganized arrangement of germ cells (Figure 3D) and rare, intraluminal, multinucleated spermatids. Few mature and immature spermatids were within the epididymal ductal lumina.

Swabs of urine and uterine fluid were submitted to WADDL for aerobic and anaerobic bacterial cultures. No bacteria were identified on aerobic and anaerobic bacterial cultures of the submitted swabs of urine and uterine fluid or on histologic examination of the submitted tissues.

### 2.4. Clinical outcome

At the time of publication (9 months following gonadohysterectomy), the patient is doing well clinically at home. Since recovering from surgery, the patient's clinical symptoms have resolved. No signs of hematuria have been observed at home. Since recovering from surgery, the patient's clinical symptoms have resolved (Supplementary Figure 1).

## 3. Discussion

Persistent Müllerian duct syndrome in a dog is a disorder of sex development due to mutations in the AMH receptor, which prevents regression of the Müllerian duct and leads to the persistence of a uterus masculinus or other rudimentary reproductive structures (15). Affected animals commonly show unilateral or bilateral cryptorchidism, and they can be fertile if at least one testis is in the scrotum (14). They usually present with a uterus, oviducts, cervix, and cranial vagina and tend to develop urinary tract infections, prostatitis, cystic endometrial hyperplasia, pyometra, and testicular tumors (2, 15, 25–31). Owing to this patient's normal male phenotype and the only clinical sign being intermittent bloody preputial discharge, PMDS was not initially considered a differential diagnosis, even though this patient was a mix with miniature Schnauzer, a breed consistent with the well-known inherited recessive disorder (15).

The differential diagnoses for hematuria and preputial serosanguinous discharge in dogs include balanitis, balanoposthitis, penile tumors, penile/preputial mating-induced trauma, prostatitis, and urinary tract infections. This patient was referred due to a possible prostatic cyst and hyperplasia, which were treated with antibiotics and the 5α-reductase inhibitor finasteride. This diagnosis was not confluent with the patient's age; in fact, prostatic hyperplasia and cysts are typical of adult intact males of 4 years of age and older. Prostatitis was excluded because he did not show pain on transrectal palpation or respond to antibiotics. Additional evaluations by ultrasonography identified the cystic structure at the level of the prostate reported by the rDVM. However, it was extending cranially and ventrally toward the inguinal rings. The CT evaluation was fundamental in obtaining a strong suspicion of an enlarged fluid-filled uterus masculinus (Figure 1, Supplemental Video 1). Mutation in the AMH receptor gene was confirmed in this dog by genetic testing, resulting in PMDS. The diagnosis of PMDS, however, did not explain the intermittent hematuria and preputial serosanguinous discharge. Exploratory laparotomy aided in the explanation of these findings. The persistent Müllerian ducts were filled with urine due to a patent and enlarged utricle connecting the cranial vagina and the prostatic urethra. This likely led to ulceration and inflammation of the uterus masculinus and the ultimate excretion of blood in the urine. This case is unique because, although Meyers–Wallen et al. described a connection between the cranial vagina and the prostatic urethra in their Miniature Schnauzer breeding colony (15), there are no existing reports of urometra/hematometra, patent prostatic utricle, and hematuria in an intact male dog.

This case also provided a unique opportunity for microscopic spermatozoa and histologic testicular analysis of a non-purpose-bred dog with PMDS. As presented in the cases by Breshears and Peters (14) and Wu et al. (32), the patient here demonstrated complete spermatogenesis on histologic analysis. Quantitative analysis of the seminiferous tubule in this patient was also consistent with their reports that when dogs with PMDS demonstrate spermatogenesis, it is often reduced when compared to normal dogs. This patient's spermatogenic potential demonstrates that the findings of Wu et al. (32) are not exclusive to purpose-pred PMDS pedigree dogs but may also be observed in a normal clinical setting.

Testosterone, inhibin B, progesterone, and AMH were evaluated, but no clear explanation existed for their concentrations. The low testosterone level, usually consistent with cryptorchid males, was surprising since the dog had descended testicles of normal size and consistency. The low testosterone level was consistent with the histologic finding of incomplete spermatogenesis and the low number of motile spermatozoa in the semen extracted from the epididymis. A possible explanation for the testicular degeneration and poor sperm production is that the tip of both uterine horns filled with urine was inside the scrotum lateral to the testes, altering their thermoregulation and normal environment. Hydrocele, an accumulation of fluid in the vaginal space surrounding the testicles, commonly causes testicular degeneration and subsequent poor sperm production. Regarding the other hormones evaluated in this case, even though reference ranges for dogs are preliminary, we can conclude that AMH concentrations were higher than an intact male dog of the same age.

In human medicine, there are many more reports of PMDS (1), where 85% of the cases are due to mutations of the AMH or AMHRII gene and 15% are unknown and could be related to abnormalities of the urogenital sinus unrelated to the AMH physiology (33). The condition presented here may be a combination of PMDS and a malformation of the urogenital region. The presence of Müllerian ducts and an enlarged prostatic utricle has been reported in boys (19, 34) but, to our knowledge, has never been described in veterinary medicine.

The presented case is novel in its presentation and subsequent treatment. Prior to this case, bilateral spermatogenesis had only been documented in PMDS purpose-bred dogs (32). Subsequently, hematuria has been commonly reported in people with PMDS (17, 19, 21, 34); however, only one other case has been reported in the canine (11). To our knowledge, our case also represents the only confirmed case of PMDS associated with an enlarged prostatic utricle in a dog. While previous reports required cystotomy- or cystoscopy-assisted visualization of the prostatic utricle/urethral communication (24), we demonstrated the utility of intraoperative fluoroscopic guided contrast cystourethrogram for minimally invasive prostatic utricle confirmation. Moreover, we demonstrated a complete resolution of lower urinary tract signs, including hematuria with gonadohysterectomy and direct prostatic utricle occlusion, contrary to a previous similar case report of undefined urine-filled prostatic cysts (24).

## Data availability statement

The raw data supporting the conclusions of this article will be made available by the authors, without undue reservation.

## Ethics statement

Written informed consent was obtained from the participant/patient(s) for the publication of this case report.

## Author contributions

PW, KM, YA, and MC contributed to the writing of the manuscript and literature review. EG and JR assessed the gross and histopathologic findings and revised the description of the histopathologic findings in the manuscript. TO contributed to the critical revision of the manuscript, assisted with the surgery on the patient, and managed the clinical case. All authors contributed to the final review, article, and approved the submitted version.

## Supplementary material

Supplementary Figure 1 Graphical abstract of a unique case of persistent Müllerian duct syndrome and urometra via an enlarged prostatic utricle in a dog with complete spermatogenesis. A 1-year-old Miniature Schnauzer cross, which was phenotypically male with descended testicles, was presented for hematuria. Advanced imaging and a genetic test confirmed urometra and homozygote mutations on *AMHRII*, respectively. Surgical treatment with gonadohysterectomy and prostatic utricle occlusion. Histologic evaluation confirmed complete spermatogenesis in this persistent Müllerian duct syndrome case. Created with BioRender.com.

Supplementary Video 1 Axial, coronal, and sagittal abdominal CT post-contrast scan images with a slice thickness of 3.0 mm. The uterus and caudally descending uterine horns are dorsal to the urinary bladder and ventral to the colon in the caudal abdomen.

Supplementary Video 2 An intraoperative fluoroscopic contrast cystourethrogram demonstrates the communication between the prostatic urethra and the vagina at the level of the colliculus seminalis.
