# Supplementary figures and images for: Case report: Persistent Müllerian duct syndrome and enlarged prostatic utricle in a male dog
Source: Front Vet Sci. 2023 Jul 4;10:1185621. doi: 10.3389/fvets.2023.1185621 (PMC10352618; doi:10.3389/fvets.2023.1185621)

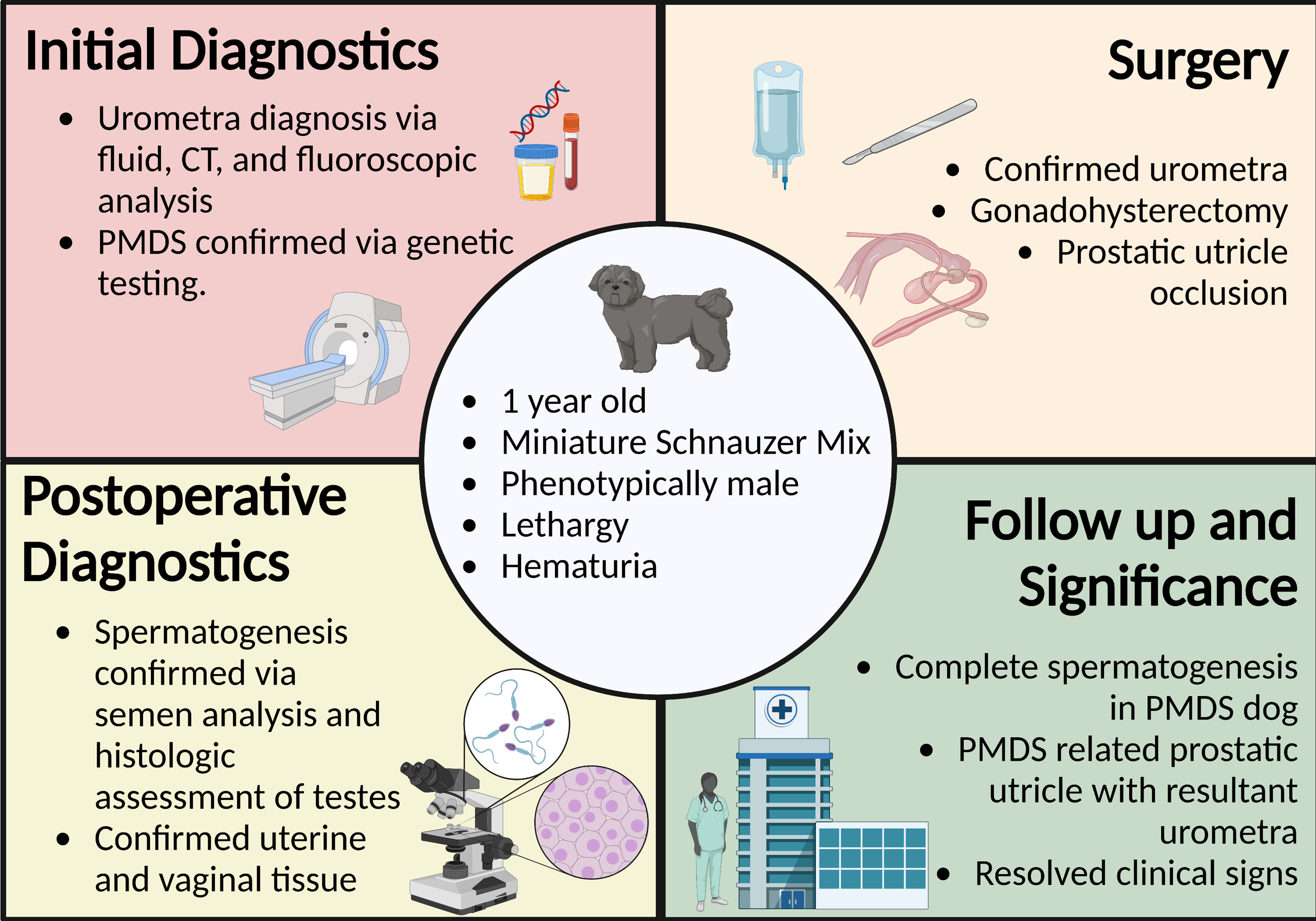

Supplement: Supplementary Figure 1 — Graphical abstract of a unique case of persistent Müllerian duct syndrome and urometra via an enlarged prostatic utricle in a dog with complete spermatogenesis. A 1-year-old Miniature Schnauzer cross, which was phenotypically male with descended testicles, was presented for hematuria. Advanced imaging and a genetic test confirmed urometra and homozygote mutations on AMHRII, respectively. Surgical treatment with gonadohysterectomy and prostatic utricle occlusion. Histologic evaluation confirmed complete spermatogenesis in this persistent Müllerian duct syndrome case. Created with BioRender.com. [file Image_1.PNG]
